# The FLS (Fatty liver Shionogi) mouse reveals local expressions of lipocalin-2, CXCL1 and CXCL9 in the liver with non-alcoholic steatohepatitis

**DOI:** 10.1186/1471-230X-13-120

**Published:** 2013-07-23

**Authors:** Toshihisa Semba, Motoi Nishimura, Satomi Nishimura, Osamu Ohara, Takayuki Ishige, Sayaka Ohno, Ken Nonaka, Kazuyuki Sogawa, Mamoru Satoh, Setsu Sawai, Kazuyuki Matsushita, Fumio Imazeki, Osamu Yokosuka, Fumio Nomura

**Affiliations:** 1 Department of Molecular Diagnosis, Graduate School of Medicine, Chiba University, 1-8-1 Inohana, Chuo-ku, Chiba 260-8670, Japan; 2 Clinical Proteomics Research Center, Chiba University Hospital, Chiba, Japan; 3 Department of Human Genome Research, Kazusa DNA Research Institute, 1-8-1 Inohana, Chuo-ku, Chiba 260-8670, Japan; 4 Division of Laboratory Medicine, Chiba University Hospital, 2-6-7 Kazusa-kamatari, Kisarazu, Chiba 292-0818, Japan; 5 Department of Medicine and Clinical Oncology, Graduate School of Medicine, Chiba University, 1-8-1 Inohana, Chuo-ku, Chiba 260-8670, Japan

**Keywords:** Adipokine, Non-alcoholic steatohepatitis, Chemokine, Immunohistochemistry, Pathogenesis

## Abstract

**Background:**

Nonalcoholic fatty liver disease (NAFLD) encompasses a wide spectrum of diseases, ranging from simple steatosis to nonalcoholic steatohepatitis (NASH), which carries a significant risk of progression to cirrhosis and hepatocellular carcinoma. Since NASH is a progressive but reversible condition, it is desirable to distinguish NASH from simple steatosis, and to treat NASH patients at an early stage. To establish appropriate diagnosis and therapy, the pathological mechanisms of the disease should be elucidated; however, these have not been fully clarified for both NASH and simple steatosis. This study aims to reveal the differences between simple steatosis and NASH.

**Methods:**

This study used fatty liver Shionogi (FLS) mice as a NASH model, for comparison with dd Shionogi (DS) mice as a model of simple steatosis. Genome-wide gene expression analysis was performed using Affymetrix GeneChip Mouse Genome 430 2.0 Array, which contains 45101 probe sets for known and predicted genes. Quantitative reverse transcription polymerase chain reaction (qRT-PCR) and immunohistochemistry were used to investigate gene expression changes and protein localizations.

**Results:**

DNA microarray analysis of the liver transcriptomes and qRT-PCR of both types of mice revealed that LCN2, CXCL1 and CXCL9 mRNAs were overexpressed in FLS mouse livers. Immunohistochemistry showed that CXCL1 protein was mainly localized to steatotic hepatocytes. CXCL9 protein-expressing hepatocytes and sinusoidal endothelium were localized in some areas of inflammatory cell infiltration. Most interestingly, hepatocytes expressing LCN2, a kind of adipokine, were localized around almost all inflammatory cell clusters. Furthermore, there was a positive correlation between the number of LCN2-positive hepatocytes in the specimen and the number of inflammatory foci.

**Conclusions:**

Overexpression and distinct localization of LCN2, CXCL1 and CXCL9 in the liver of fatty liver Shionogi mice suggest significant roles of these proteins in the pathogenesis of NASH.

## Background

Nonalcoholic fatty liver disease (NAFLD) is the most common etiology of chronic liver disease in the United States and other developed countries [1]. It is closely associated with metabolic syndrome, which consists of a constellation of insulin resistance, central obesity, hypertension and dyslipidemia [2]. For this reason, NAFLD has been regarded as a hepatic manifestation of metabolic syndrome [3]. NAFLD includes a spectrum of liver diseases ranging from simple hepatic steatosis (SS) to nonalcoholic steatohepatitis (NASH) [4]; the latter is known to increase the risk of liver cirrhosis and hepatocellular carcinoma [5]. Individuals with simple steatosis rarely develop significant disease, whereas nearly 20% of those with NASH progress to end-stages of liver disease [6,7]. Evidence that cirrhosis and hepatocellular carcinoma are more likely to develop in individuals with NASH rather than those with SS suggests that NASH is a more serious form of liver injury [6,8,9].

NASH is histologically similar to alcoholic steatohepatitis, with the presence of macrovesicular steatosis, mixed inflammatory cell infiltration of the lobules, ballooning degeneration and necrosis of hepatocytes, Mallory body formation, and perisinusoidal fibrosis [7]. Although the pathogenesis of NASH remains unclear, hepatic steatosis is the setting for NASH, with multiple ‘hits’ , such as oxidative stress, inevitably leading to this progressive form of inflammatory liver disease [9,10]. Furthermore, the role of the gut microbiota and other new mechanisms in the development of NAFLD have recently been discovered [11]. An abnormality in the balance of secretion of cytokines and adipokines is thought to be one such multiple ‘hit’ [10,12]. Therefore, investigation of cytokines/adipokines has the potential to elucidate the pathogenesis of NASH and to lead to the discovery of relevant biomarkers. On the other hand, cytokines and adipokines are also affected by inflammation and in simple obesity. Thus, the pathological significance of the localization of these molecules in NASH livers remains unclear. Further detailed investigation is needed to develop cytokines and adipokines as NASH markers. From an ethical perspective [13], human liver biopsy cannot be performed casually because of its invasiveness, which has contributed to the delay in clarification of the pathological details of NASH. On the other hand, there are inbred experimental mice in which the development of spontaneous fatty liver is a result of specific gene mutations, such as ob/ob, db/db, fat/fat and tub/tub, although these animals do not develop spontaneous NASH [14-17]. In recent years, the inbred “fatty liver Shionogi” (FLS) mouse model has been developed [18]; these animals develop fatty livers in a setting of insulin resistance under normal environmental conditions, which finally progresses to concurrent hepatocellular carcinoma via steatohepatitis. These mice have been used as animal models to compare the pathophysiology of NASH with that of SS [18-22]. Therefore, in this study, as a first step toward development of novel NASH biomarkers, these mice and DS mice, which are a sister strain of FLS, were used for evaluation and comparison of their hepatic transcriptomes.

## Methods

### Animals

FLS and DS mice (9 each), aged 12 weeks, were obtained from Aburabi Laboratories, Shionogi Research Laboratories (Shionogi and Co., Ltd, Shiga, Japan).The FLS mice were derived from the C line of an outbred ddN colony [23] and were established as an inbred strain by full-sib mating. The DS mice were derived from the A line of an outbred ddN colony and were also established as an inbred strain by full-sib mating [23]. The DS strain does not manifest liver abnormalities, including NASH phenotype, [18] and was, thus, used as a reference strain in this experiment. The mice, kept in separate cages to prevent fighting, were housed in an air-conditioned room at 23 ± 3°C and 50 ± 20% relative humidity, with a 12 h light/12 h dark cycle (6:00–18:00 hours). All mice were given a standard pellet diet (CE-2; Clea Japan Inc, Tokyo, Japan), and free access to water. The physiological and biochemical parameters of the mice are summarized in Table 1. All experiments were carried out in accordance with the Animal Experimentation Guidelines of Chiba University and the study protocol was approved by the Ethical Committee of Graduate School of Medicine, Chiba University.

**Table 1:** Physiologic and biochemical parameters in FLS and DS mice at 19 weeks of age

**Variable**	**FLS**		**DS**		***P*value**
Food intake (g/day)	4.0	±0.1	4.1	±0.1	NS
Body weight (g)	33.0	±6.2	45.4	±3.7	<0.001
Tissue weights					
Liver (g)	1.65	±0.44	1.85	±0.33	NS
Liver (g)/BW(g) × 100	4.96	±0.75	4.06	±0.53	<0.01
Serum					
AST (IU/L)	77	±20	51	±18	NS
ALT (IU/L)	84	±50	23	±3	<0.05
T-Cholesterol (mg/dL)	110	±5	107	±14	NS
Triacylglycerol (mg/dL)	103	±42	85	±32	NS

Abbreviations: *AST* aspartate aminotransferase, *ALT* alanine aminotransferase, *NS* not significant.

Mean ± SD. (n = 3 to 9)

Mann–Whitney U-test for comparisons between groups.

### Sample collection

After administration of pentobarbital (Nembutal, Abbott Laboratories, Chicago, IL, USA) for euthanizing the animals, saline perfusion was performed to remove organ blood, and the livers were immediately removed, weighed and sliced; one portion was fixed in a 15% neutral formalin buffer solution, pH 7.4 (Wako Pure Chemicals, Tokyo, Japan) and embedded in paraffin for histological analysis, and the rest were snap frozen in liquid nitrogen and stored at −80°C for subsequent analysis.

### Light microscopy

Fixed tissues embedded in paraffin were sectioned and, after appropriate standard treatments, were stained with hematoxylin and eosin (H&E). The remaining formalin-fixed liver tissues were processed for oil red O and Masson’s Trichrome staining. Assessment of NAFLD specimens was based on the criteria described by Matteoni [7].

### Microarray data analysis

**Figure 1:**
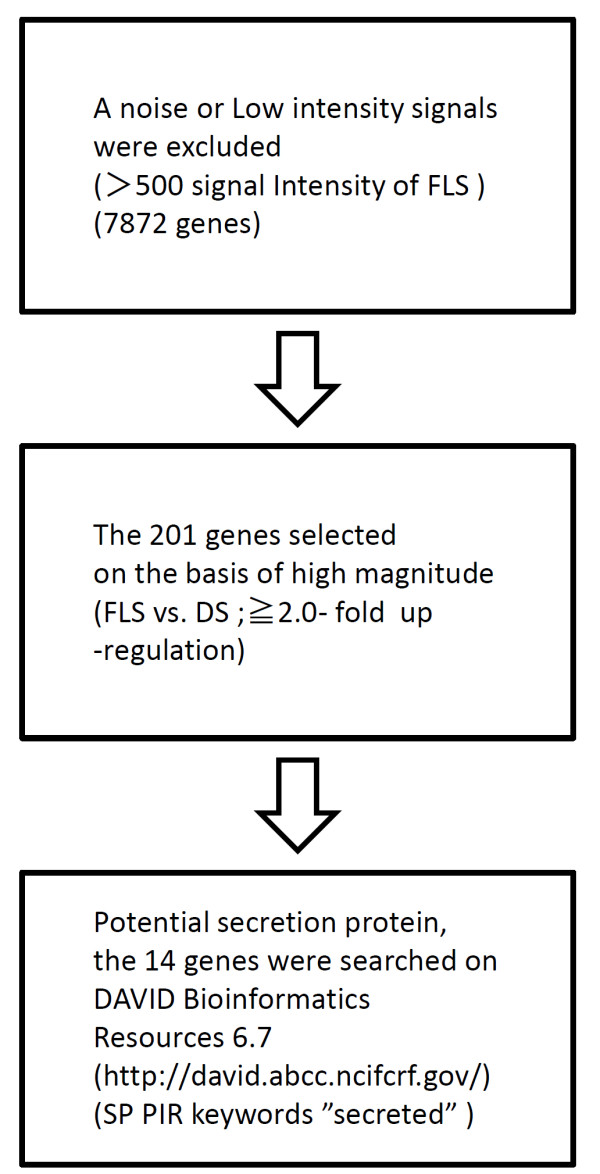
**Strategy for selection of potential serum biomarkers.** The potential serum biomarkers were searched for in the copy of the full microarray data set has been deposited in GEO Express (accession no. GSE45624). Biomarkers were selected on basis of higher the gene expression ratio and potential secretion protein.

Total RNA was extracted from the livers of 3 FLS and 3 DS mice at 19 weeks of age using RNeasy mini kits (QIAGEN Inc., Valencia, CA, USA). RNA from each strain was pooled and subjected to messenger RNA expression profiling using a microarray (Mouse genome 430 2.0 array, Affymetrix, Santa Clara, CA, USA). A copy of the full microarray data set has been deposited in GEO Express (accession no. GSE45624) and were normalized and analyzed using the MBEI (PM-only) algorithm [24]. The 14 candidate NASH biomarkers (Table 2) having larger expression differences between FLS livers and DS livers were chosen according to criteria shown in Figure 1. Among these candidates, we focused on genes encoding immune response proteins (such as chemokines and adipokines), because the number of hepatic inflammatory cells differs markedly between NASH and SS, being minimal in SS and significantly up-regulated in NASH [25].

**Table 2:** Microarray identified genes differentially expressed in FLS and DS mice at 19 weeks of age

**Genebank accession**	**Gene symbol**	**Gene description**	**Fold change**	**Signal intensity** **FLS**	**DS**
NM_011016	Orm2	orosomucoid 2	7.0	3262.0	466.7
NM_011819	Gdf15	growth differentiation factor 15	4.6	3100.4	674.6
AL385586	Ang	angiogenin, ribonuclease, RNase A family, 5	4.5	1512.2	338.5
X14607	Lcn2	lipocalin 2	4.3	1439.9	331.8
NM_010406	Hc	hemolytic complement	3.2	7329.8	2321.0
NM_008599	Cxcl9	chemokine (C-X-C motif) ligand 9	28	982.1	350.7
AW550625	Col3a1	collagen, type III, alpha 1	2.8	1409.3	507.4
BB554288	Cxcl1	chemokine (C-X-C motif ) ligand 1	2.6	3938.2	1515.6
AKO11784	lgfbp2	insulin-like growth factor binding protein 2	2.5	12400.5	4979.9
BC005569	Rnase4	ribonuclease, RNase A family 4	2.4	7907.8	3254.3
BM122014	Gm6484	predcited gene 6484	2.4	1462.2	605.6
BC025830	Pm20d1	peptidase M20 domain containing 1	2.3	3390.0	1471.5
NM_011315	Saa3	serum amyloid A 3	2.2	1242.3	553.2
BF227507	Col1a2	collagen, type l, alpha 2	2.0	632.6	315.6

### Quantitative real-time polymerase chain reaction

Total RNA was extracted from all mouse livers using an RNeasy Mini kit and DNeasy Tissue kit (QIAGEN Inc., Valencia, CA, USA). cDNA was synthesized from total RNA with the first-strand cDNA Synthesis kit for RT-PCR (Roche, Mannheim, Germany). Quantitative (q) PCR was carried out on the LightCycler 480 System (Roche Diagnostics, Rotkreuz, Switzerland) using the LightCycler 480 SYBR Green I Master (Roche Diagnostics). The PCR primers used in this study are listed in Table 3. Relative levels of mRNA in each sample were expressed after normalization to β-actin as a housekeeping gene.

**Table 3:** PCR primers used in this study

**Primer**	**Forward**	**Reverse**	**Tm value (°C)**	**Product size (bp)**
Lcn2	CTGTCGCTAGATCAGAAC	TGTACCTGAGGATACCTGTGC	60	89
Cxcl1	AGCCACACTCAAGAATGGTCG	TTACTTGGGGACACCTTTTAG	60	200
Cxcl9	CCGAGGCACGTCCACTTACA	TCTAGGTTTGATCCCGTTC	60	117
β-actin	CATCCGTAAAGACCTCTATGCCAAC	ATGGAGCCACCGATCCACA	60	171

### Immunohistochemistry

The primary antibodies used in this study are listed in Table 4. Immunohistochemistry was performed on both paraffin-embedded and frozen sections according to standard protocols. The LSAB + system (DAKO Japan, Kyoto, Japan) was used to visualize the tissue antigens lipocalin-2 (LCN2), CXCL1 and CXCL9, according to the manufacturer’s instructions. Histofine Simple Stain MAX-PO (R) (Nichirei, Tokyo, Japan) and DAB solution (Dako) were used to visualize CD3, F4/80 and the neutrophil marker, according to the manufacturer’s instructions.

**Table 4:** Primary Antibodies used in this study

**Antibody**	**Catalog number**	**Host**	**Cell identified**	**Source**	**Tissue fixation**	**Titre**
Lcn2	AF1857	Goat	-	R&D Inc. *	Formalin	1:50
Cxcl1	AF-453-NA	Goat	-	R&D	Formalin	1:100
Cxcl9	AF-492-NA	Goat	-	R&D	Formalin	1:100
Neutrophil Marker	sc-59338	Rat	Neutrophils	Santa Inc. †	Formalin	1:200
CD3	sc-101442	Rat	Lymphocytes	Santa	Frozen	1:500
F4/80	sc-59171	Rat	Macrophages	Santa	Formalin	1.50

*R&D Systems Inc., Minneapolis, Minnesota, USA. †Santa Cruz Biotechnology Inc., Santa Cruz, California, USA.

### Morphometric analysis

The number of LCN2-positive hepatocytes and inflammatory foci (which included lymphocytes, macrophages and neutrophils) were evaluated by computer-aided morphometry, as reported previously [26]. Data were expressed as mean cell count per 1 cm^2^ area of tissue.

### Western blot analysis of LCN2

Protein extraction and western blotting were performed as described previously [27] Anti-lipocalin-2 goat polyclonal antibody (R&D Systems, Inc., AF1857) diluted 1:250 and anti-β-actin goat polyclonal antibody (Santa Cruz Biotechnology Inc., Santa Cruz, CA, USA) diluted 1:500 in blocking buffer were used as primary antibodies.

### Statistical analysis

Statistical analyses were performed using nonparametric tests (R, version 2.13.2) [28]. Differences between the two groups were evaluated by the Mann–Whitney U-test. Associations between two parameters were evaluated by Spearman rank correlation coefficients (Spearman’s ρ). *P* values < .05 were considered significant.

## Results

### Histopathological observations

**Figure 2:**
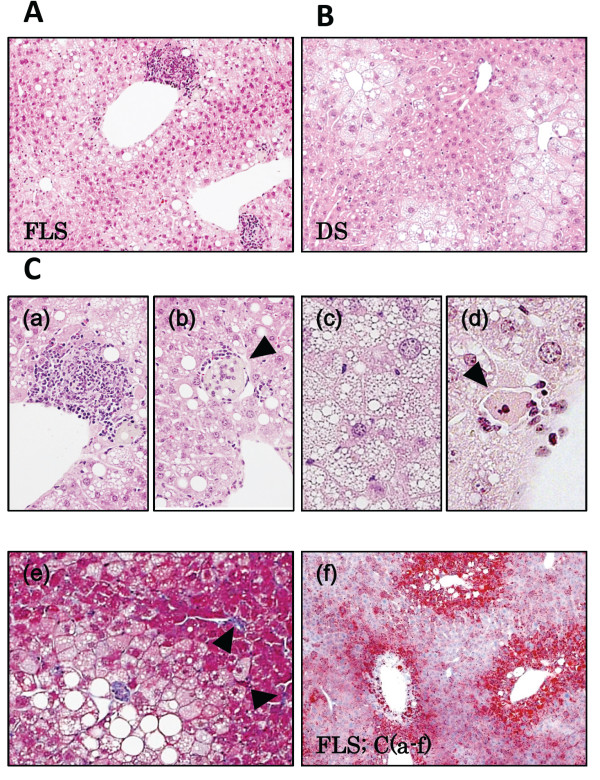
**Microscopic findings in FLS and DS mouse livers at 19 weeks of age. (A)** FLS mice, **(B)** DS mice, **(C)** Microscopic characteristics of FLS mice at 19 weeks: **(a)** Focus of inflammation; **(b)** Balloon cells; **(c)** Foamy cell (arrowhead); **(d)** Acidophilic body (arrowhead); **(e)** Fibrosis (arrowheads); **(f)** Oil Red O staining. **(A-C)** Hematoxylin and Eosin staining, original magnifications: A, B, ×100; C-a, c, ×200; C-b, d, ×400. **(C-e)** Masson’s Trichrome staining; original magnification, ×200. **(C-f)** Original magnification, ×60.

Large and small vacuoles were observed in H&E stained liver specimens from both FLS and DS mice (Figure 2A, B). Fat droplets in FLS livers were larger than those in DS mice, and mild phagocytic infiltration of the liver lobules was found in 8 FLS animals (89%), but in none (0%) of the DS group animals (Figure 2C;a) (Table 5). Balloon-like swelling of hepatocytes (a finding suggestive of hepatocyte volume impairment due to excess fat accumulation) was noted in 5 DS animals (56%) and in 8 FLS animals (89%) (Figure 2C;b). Foamy appearance and eosinophilic necrosis were noted in FLS mice (Figure 2C;c, 2C;d, 3D). Mild fibrosis of the liver was observed by Masson’s Trichrome staining in several FLS mice (Figure 2C;e). Staining by Oil red O confirmed accumulation of neutral fat in the vacuoles (Figure 2C;f). Therefore, these experimental 19-week-old FLS and DS mice were adopted as the NASH and fatty liver models, respectively.

**Table 5:** Histology of various FLS and DS mouse tissues

**Strain**	**Age (weeks)**	**No. of mice**	**Incidence**			
			**Normal(%)**	**Fatty liver(%)**	**Ballooning hepatocyto(%)**	**Steatohepatitis(%)**
FLS	19	9	0(0)	9(100)	8(89)	8(89)
DS	19	9	1(11)	8(89)	5(56)	0(0)

### Varying degrees of inflammatory cell infiltration in NASH mice

**Figure 3:**
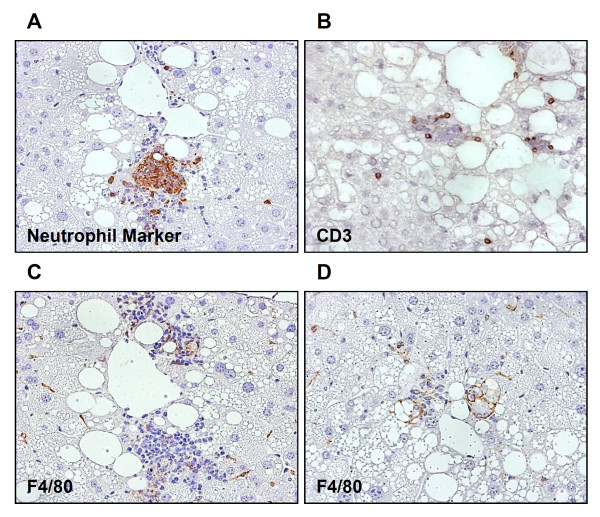
**Immunohistochemistry of hepatic inflammatory focus in FLS mice at 19 weeks of age.** The inflammatory infiltration consisted of various immune cells. Immunostaining for **(A)** neutrophils, **(B)** lymphocytes, and **(C, D)** macrophages. Original magnification, ×400. Inflammatory cells mainly consisted of neutrophils and lymphocytes.

Clusters of hepatic inflammatory cells were identified in the 19-week-old FLS mice by immunohistochemical staining; neutrophil-marker-positive (Figure 3A), CD3-positive (Figure 3B) and F4/80-positive (Figure 3C, D) cells coexisted in these liver samples. A larger number of positive reactions to neutrophil markers than to F4/80 or CD3 were found.

### Identification of altered gene expression in NASH mice

As mentioned, we found marked fatty changes and inflammatory cell accumulations in FLS livers. We also found that these inflammatory cells were heterogenous. They mainly consisted of neutrophils and lymphocytes. Therefore, we focused on inflammation-related molecules, especially adipokines and chemokines, as possible NASH biomarker candidates. Messenger RNA expression profiling revealed that adipokines (LCN2) and chemokines (CXCL1, CXCL9) were among the genes upregulated in FLS livers (Table 2).

### Detection of LCN2 by qRT-PCR and immunohistochemistry

**Figure 4:**
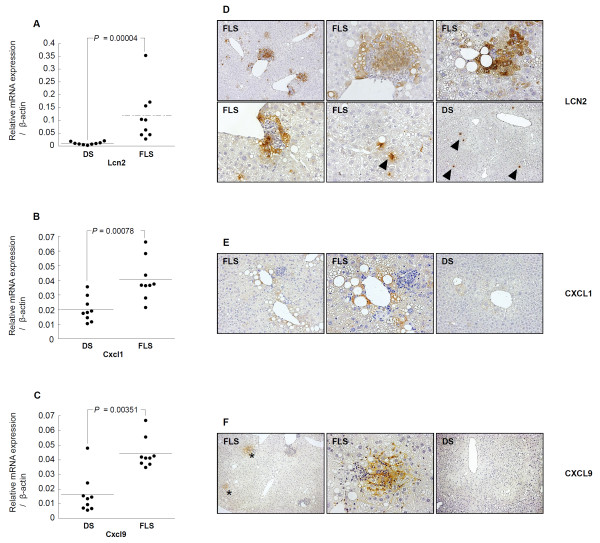
**Confirmation of changes in the expression of several genes and representative findings of immunohistochemical staining in FLS and DS mice livers at 19 weeks of age.** Real-time PCR analysis of liver tissue from mice (n = 18) revealed altered gene expression for **(A)** LCN2, **(B)** CXCL1, and **(C)** CXCL9, as detected by microarray analysis. Analysis results of the three genes were in directional concordance with microarray results and were statistically significantly upregulated in FLS compared with DS mice (p < 0.05). Differences between the FLS and DS groups were assessed by the Mann–Whitney U-test. **(D)** Hepatocytes strongly positive for LCN2 were aggregated close to most inflammatory foci, whereas DS mice hepatocytes were scarcely stained. LCN2 protein was also expressed in some mononuclear cells in both types of mice (arrowheads). **(E)** CXCL1 showed strong staining in some steatotic hepatocytes of FLS mice compared with faint staining in DS mice. **(F)** CXCL9 showed strong staining in the sinusoidal endothelium and hepatocytes present in the vicinity of some inflammatory cell clusters in FLS mice (asterisks), whereas DS mice hepatocytes were scarcely stained.

**Figure 5:**
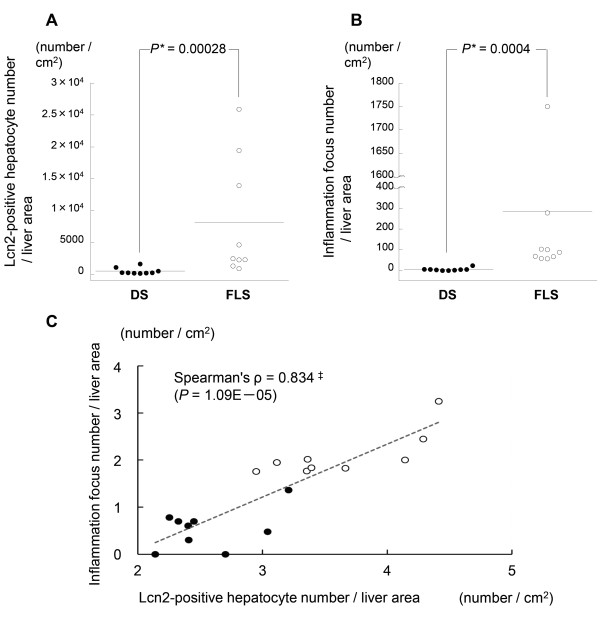
**Quantification of LCN2 and inflammation in hepatic specimens from 19-week-old mice. (A)** LCN2 protein expression: number of positive hepatocytes per cm^2^; **(B)** Number of inflammatory foci per cm^2^; **(C)** Correlation between LCN2 protein-expressing hepatocytes (log scale) and number of inflammatory foci (log scale) (n = 19). *Mann–Whitney U-test. ^‡^Spearman rank correlation coefficient; solid circles, DS; open circles, FLS.

Real-time PCR was used to determine the amounts of LCN2 gene expression in the livers of 19-week-old mice; expression was found to be increased significantly in FLS compared to DS mice (0.118 ± 0.1015 vs. 0.01 ± 0.005, P = 0.00004) (Figure 4A). Western blot analysis confirmed that LCN2 protein was increased in FLS as compared to DS mice (Additional file 1: Figure S1). Strongly positive immunohistochemical staining was observed in parenchymal hepatic cell cytoplasm, especially those in the vicinity of inflammatory cells (Figure 4D). Furthermore, the number of LCN2-positive cells per unit area, as determined by morphometric analysis, was significantly greater in FLS as compared to DS mice (P = 0.00028) (Figure 5A). Similarly, evaluation of the number of foci of inflammatory cell clusters in serial sections by H&E staining revealed that the number was significantly greater in FLS as compared to DS mice (P = 0.0004) (Figure 5B). There was a positive correlation between the number of LCN2-positive hepatic cells per unit area and the number of inflammatory cell clusters in both groups of mice (Spearman’s ρ = 0.834; P = 1.09E-05) (Figure 5C).

### Detection of CXCL1 by qRT-PCR and immunohistochemistry

Real-time PCR revealed that CXCL1 mRNA expression was increased significantly in FLS as compared to DS mice (0.041 ± 0.014 vs. 0.02 ± 0.001, P = 0.00078) (Figure 4B). Furthermore, strongly positive CXCL1 staining was found in steatotic hepatocytes; staining intensities tended to be stronger in FLS as compared to DS mice (Figure 4E).

### Detection of CXCL9 by qRT-PCR and immunohistochemistry

The amount of CXCL9 mRNA expression was found to be increased significantly in FLS as compared to DS mice (0.044 ± 0.01 vs. 0.016 ± 0.001, P = 0.00351) (Figure 4C). Furthermore, strongly positive CXCL9 staining was found in the cytoplasm of hepatic parenchymal cells in the vicinity of inflammatory cells, especially in the sinusoidal endothelium, although some of these cells were completely negative for CXCL9 (Figure 4F). This positive reactivity was greater in FLS as compared to DS mice.

## Discussion

NASH is a progressive disease; hence, discriminating between benign fatty liver and NASH is very important for prediction of its prognosis. Although establishment of appropriate diagnosis and therapy requires elucidation of the pathological mechanisms of this condition, these have not been fully clarified for NASH and NAFLD.

NAFLD is a complex disease with no simple answers [12]. However, epigenetic mechanisms of nuclear chromatin remodeling and oxidative stress, in particular, are increasingly being recognized as crucial factors in the pathophysiology of NAFLD [11]. Oxidative stress is associated with the secretion of inflammatory biomolecules, including cytokines. Indeed, during the chronic inflammation present in NASH, a systemic release of pro-inflammatory mediators from the liver occurs [11]. Therefore, it is likely that detailed study of the dynamic state of cytokine and adipokine levels in the NASH liver may lead to deeper understanding of the disease, as well as development of better disease markers.

The present study was a search for candidate NASH markers through comprehensive gene expression analysis of livers from NASH- and SS-model mice. Interestingly, one adipokine (LCN2) and two chemokines (CXCL1, CXCL9) were included in the top 14 genes identified as candidate NASH markers (Table 2). Inflammatory cells prominent in FLS mouse livers mainly consisted of neutrophils and lymphocytes (Figure 3). Therefore, we focused on expression levels of lymphokines and chemokines in this study. In terms of distribution in the hepatic lobule, focal expressions of LCN2 were found in hepatic parenchymal cells in the vicinity of inflammatory cell clusters by immunohistochemical staining techniques (Figure 4D), and correlations were found between the number of inflammatory clusters and number of LCN2-positive cells in the NAFLD (NASH and SS) mice (Figure 5C).

LCN2 was originally reported as a glycoprotein (NGAL) with a molecular weight of 25 kDa that is present in leukocytes [29]. Since then, it has been reported that it is expressed in the liver upon administration of endotoxin [30] and that it is produced in hepatic cell lines in response to exposure to hydrogen peroxide [31]. In NASH, the involvement of endotoxin, oxidative stress and cytokines have been indicated as some of the multiple ‘hits’ [11,12]; this is compatible with the current findings of LCN2 expression in NASH livers (Figure 4A, Additional file 1 Figure S1). Furthermore, expression of LCN2 protein was prominently observed in hepatic parenchymal cells in the vicinity of inflammatory cells (Figure 4D). This suggests that the hepatocytes in this microenvironment are subjected to stresses, such as oxidative stress and exposure to cytokines. In recent years, it has been reported that LCN2 plays a role in protecting hepatic cell lines from the toxicity of hydrogen peroxide [32]. When combined with the current results on the focal presence of LCN2 protein in the NASH liver, it is possible that, in the pathogenesis of NASH, liver LCN2 expression is increased in response to cytotoxic stresses having origins in inflammatory cells. On the other hand, there is a report indicating prominent expression of LCN2 in the liver and visceral adipose tissues, together with elevated blood levels, in obese mice due to excessive food intake, suggesting an association between low-grade inflammation and diet-induced obesity [33]. In another study as well, prominent expression of the LCN2 protein was found in the visceral adipocytes of obese patients (with insulin resistance and elevated high-sensitivity CRP but without apparent liver injury) [34]. Thus, the results of the present study are consistent with previous studies suggesting relationships between fatty change, insulin resistance, inflammatory cell infiltration and LCN2 expression in local tissues. However, further basic or clinical studies are needed to test the pathogenic or diagnostic values of LCN2 expression.

CXCL1 is known as a chemokine for neutrophils [35]. Forced expression of the CXCL1 gene in the rat liver was accompanied by neutrophil infiltration, and led to hepatic dysfunction (elevation of aspartate aminotransferase and alanine aminotransferase) [36]. Furthermore, a correlation was found between CXCL1 protein concentration and neutrophil count in the liver of patients with alcoholic hepatitis [37]. In addition, neutrophilic infiltration of liver tissue is known to cause insulin resistance in the liver [38]. In the present study, CXCL1 protein was observed mainly in the loci of steatotic hepatocytes, the staining intensities of which tended to be stronger in FLS compared to DS mice (Figure 4E), and a large number of infiltrating neutrophils were observed in the liver of FLS mice (Figure 3). To the best of our knowledge, this study is the first to report the localization of CXCL1 protein in the NASH liver; these results, considered together with those of previous studies [35-38], suggest that CXCL1 is indeed involved in the accumulation of hepatic neutrophils in this disease and that neutrophilic infiltration may be involved in insulin resistance in FLS livers [21,22].

While CXCL1 expression is elevated by the administration of lipopolysaccharides in adipocytes co-cultured with macrophages, this is not the case when lipopolysaccharide is administered to adipocytes or macrophages alone [39]. In addition, it is reported that CXCL1 gene expression in the liver is elevated, and that the number of myeloperoxidase-positive neutrophils is increased, by provoking Fas-induced apoptosis in the livers of mice with obesity induced by a high-fat diet [40]. We observed macrophage clusters and eosinophilic necrotic cells in the NASH mouse model used in the current study (Figure 2). Furthermore, expression of CXCL1 was significantly higher in the livers of NASH mice than SS mice (Figure 4B). These results suggest the possibility that, in comparison to SS livers, interactions between a number of hepatic factors and fat deposits could be involved in the greater elevation of CXCL1 expression in NASH livers; additional studies are required to explore the mechanisms leading to hepatic CXCL1 elevation in NASH.

CXCL9 is a chemokine with a molecular weight of 14 kDa that is chemotactic for Th1 (CD4 + T lymphocytes) and NK cells, and is mediated by the CXCR3 receptor [41]. It has been reported that CXCL9 gene expression is elevated after stimulation of mouse monocytes, neutrophils and hepatocytes by interleukin γ and viral infection [42]. In the livers of HCV-infected mice, this protein was found to be expressed in hepatic parenchymal cells in the vicinities of sinuses and lymphocytes [43], and was also related to the grade of hepatic fibrosis [43,44]. In addition, it has been reported that *in vitro* experiments have suggested the possibility that migration of CD4 + T lymphocytes to hepatic parenchyma in response to CXCL9 chemotaxis is mediated by biliary epithelial cells [45]. In the current experiments, expression of this protein was found in the hepatic parenchyma and sinusoids in the vicinity of some of the inflammatory cell clusters in NASH livers (Figure 4F), consistent with previous reports; to the best of our knowledge, this is the first clear demonstration of the localization of CXCL9 protein in the NASH liver. Additionally, in this study, CXCL9 gene expression in NASH mice was greater than that in SS mice (Figure 4C). We also found CD3 positive pan-T cells in the NASH livers (Figure 3B). These results suggest the possibility that CXCL9 is involved in hepatic inflammatory cell infiltrations in NASH.

## Conclusions

**Figure 6:**
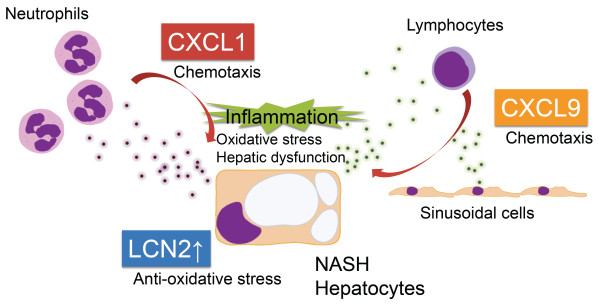
**Hypothesis of the role of LCN2, CXCL1 and CXCL9 in the pathogenesis of NASH.** Inflammatory chemokines, CXCL1 and CXCL9, are produced in the NASH liver and lead to the recruitment of inflammatory cells, such as lymphocytes and neutrophils. Expression of the CXCL1 gene is known to be accompanied by neutrophil infiltration, and to lead to hepatic dysfunction [36]. CXCL9 is a molecule that is chemotactic for lymphocytes [41]. The adipokine, LCN2, was observed in the cytoplasm of parenchymal hepatic cells, especially those in the vicinity of inflammatory cells (Figure 4D). It has been reported that LCN2 plays a role in protecting cells from the toxic effects of oxidative stress [32].

In conclusion, as a result of comparative transcriptome analysis of mice livers presenting with NASH and SS, we found that the adipokine LCN2, and chemokines CXCL1 and CXCL9 are overexpressed in NASH livers. Immunohistochemical studies revealed that these genes are related to the inflammatory process of NASH. We have schematically shown our hypothesis for their role in the pathogenesis of NASH in Figure 6. We found a correlation between hepatic LCN2 protein expression levels and inflammatory foci, suggesting that LCN2 may be a potential inflammatory marker of NASH.

## Abbreviations

NAFLD: Non-alcoholic fatty liver disease; NASH: Non-alcoholic steatohepatitis; FLS: Fatty liver Shionogi; DS: Dd Shionogi; SS: Simple hepatic steatosis; LCN2: Lipocalin-2; CXCL1: Chemokine (C-X-C motif) ligand 1; CXCL9: Chemokine (C-X-C motif) ligand 9; qRT-PCR: Quantitative real-time polymerase chain reaction

## Competing interests

The authors declare that they have no competing interests.

## Authors’ contributions

TS performed the majority of experiments and manuscript writing. MN and FN conceived the study and participated in the study design, performance, coordination and manuscript writing. SN, OO, TI and KN performed and interpreted DNA microarray data. TI participated in analyzing all qRT-PCR. SO performed biochemical tests and analyzed data. KS, MS, SS and KM provided intellectual input into many experiments. FI and OY participated in pathological interpretation. All authors read and approved the final manuscript.

## Pre-publication history

The pre-publication history for this paper can be accessed here:

http://www.biomedcentral.com/1471-230X/13/120/prepub

## Supplementary Material

Additional file 1: Figure S1 Western blot confirmation that LCN2 protein is more strongly expressed in the tissues of FLS mice (n = 3) than DS mice (n = 3). Click here for file
