# Supplementary figures and images for: The FLS (Fatty liver Shionogi) mouse reveals local expressions of lipocalin-2, CXCL1 and CXCL9 in the liver with non-alcoholic steatohepatitis
Source: BMC Gastroenterol. 2013 Jul 23;13:120. doi: 10.1186/1471-230X-13-120 (PMC3729543; doi:10.1186/1471-230X-13-120)

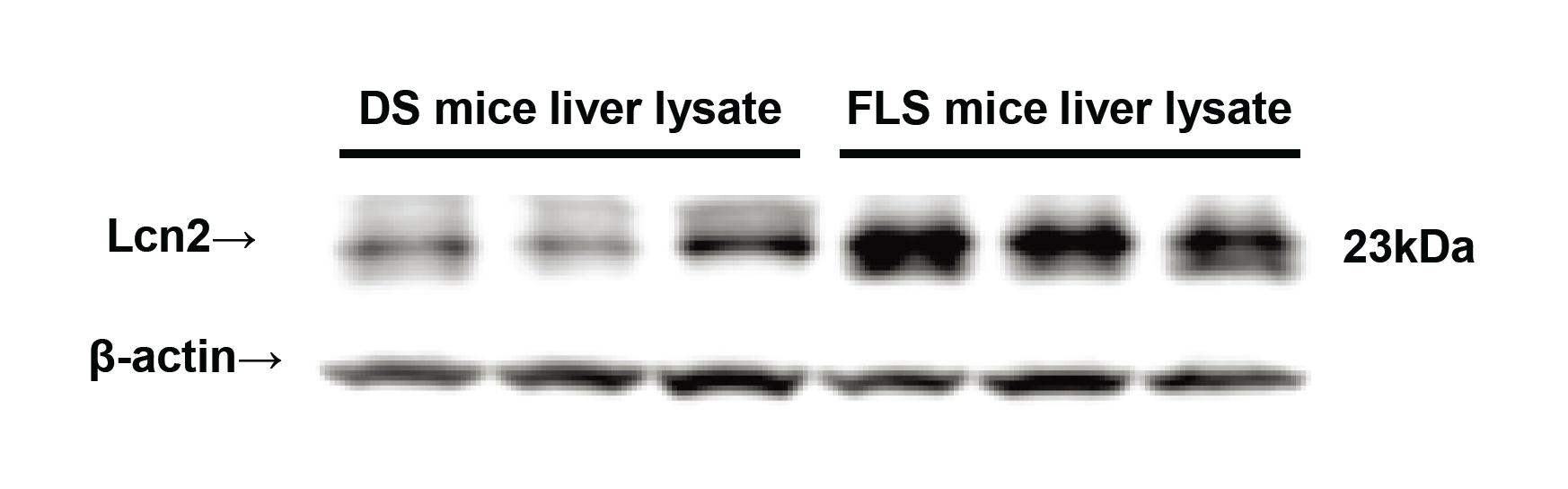

Supplement: Additional file 1: Figure S1 — Western blot confirmation that LCN2 protein is more strongly expressed in the tissues of FLS mice (n = 3) than DS mice (n = 3). [file 1471-230X-13-120-S1.tiff]
